# Indents

**Published:** 1904-10-15

**Authors:** 


### INDENTS.

☞ **Brief Articles of Technical or Scientific Value will receive notice and comment. Contributions invited.**

**Setting Inlays.** To prevent moisture reaching the inlay after seating it in place, cover with the surplus cement and firmly press a piece of tin foil over the whole. This will adhere for hours and is, of course, absolutely impervious to moisture.—C. B. Rohland in *Review*.

**Avoid Unnecessary Pain While Operating.** The comfort and welfare of the patient comes first. It is better to perform an operation twice with minimum pain than once at too great a price of suffering. This is heresy, but I was born a heretic.—Mark G. McElhinney, in *Review*.

**Temporary Filling.** Absorbent cotton saturated with cement which has been mixed to a creamy consistency makes an excellent temporary filling material. It will last for months. If only required for a short time, nearly fill the cavity with dry cotton before inserting the plug. This will facilitate the removal of the filling when it becomes necessary.—R. E. Sparks, in *International*.

**Crowning Separaled Roots.** When the two roots of a lower molar are split apart so far in the gum as to make it impracticable to draw them together, it is often advisable to use the crown as described by Dr. Goslee, that is, to band each separately, join the approximal edges of the band near the cutting edge, and place a single grinding surface over the two, thus getting the rigid support of both roots, and making it possible to cleanse the necks of each perfectly with toothpicks and antiseptic washes.—DR. HEAD.

**Natural Crown.** The Doctor had suggested that the defect in the anterior part of the mouth be made good by the adjustment of a porcelain-faced crown. The lady hesitated, then asked somewhat dubiously, "Will it look natural, Doctor?" "Natural! My dear madam, it will look so natural that you will want all the others cut off and substitutes set to match it!"

**A Hickory Tooth.** An Arkansas dentist told me that he once had a root with openings on four sides and one in the end, and that he did everything he ever heard of for it, but at length had to pull it out. He then whittled out a tooth from a piece of Mississippi River bottom scaly-bark hickory and fitted it into the socket. It took root and became firm, but during the fourth year it bore a crop of scaly bark which almost choked the patient to death and he had to pull it out.—Wm. Crenshaw.

**Preparing the Operative Field.** No one can fail to be astonished if he undertake to estimate the number of well-accredited dentists who commence filling the teeth of a new patient and never think of first cleaning and polishing them. This initial cleansing and polishing looms up as the primary point of importance, and it might serve a useful purpose if representative societies would in a formal manner declare their position in this matter. I say without hesitation that, except for the alleviation of pain of some temporary work of emergency, it ought to be considered absolute malpractice to proceed with dental operations unless this initial cleansing and polishing has first been efficiently performed.—M. L. Rhein, in *Digest.*

**Mrs. Sarah Jane White.** Mrs. Sarah Jane White, of 1622 Arch St., Philadelphia, died in her 80th year, at Rye, N. Y., where she had been visiting her daughter, Mrs. James S. McCulloh. Mrs. White was born in Wilmington, Del., Aug. 29, 1824, her maiden name being Carey, and was married in 1846 to the late Samuel S. White, founder of the business which has since been carried on by his sons under the name of the S. S. White Dental Manufacturing Company. Mrs. White is survived by two sons—J. Clarence White, of Germantown, and Samuel S. White, of Bryn Mawr—and two daughters—Mrs. Henry M. Warren, of Devon, and Mrs. McCulloh, of Rye. Another daughter, Mrs. Granville B. Haines, died a few years ago.—*Cosmos*.

**Instruct with Vigorous Speech.** I never saw mouths in such good condition as those Dr. Smith showed me in his office, but I have not been able to induce my patients to keep their mouths in such excellent shape. However, I attribute Dr. Smith's success to something more than his monthly treatment. He is vigorous and sarcastic in speech, and I believe his success lies not so much in getting his patients to come to his office every thirty days as that he impresses them with the importance of their so doing, and they would hardly dare come to him a second time unless they had been carefully and thoroughly brushing their teeth. Be this as it may, it is surely a good thing to see one's patients often, as it must increase diligence on their part.—J. N. Crouse, in *Digest*.

**To Attach Low-Fusing Body to High-Fusing Body.** 1st. Grind the facing, or other high-fused bit of porcelain, until every trace of glaze on the surface to which you wish to make a low-fusing addition is thoroughly removed.

2d. Give the ground surface a very thin coat of low-fusing body.

3d. Not only fuse, but *flow* and melt this thin layer over the prepared surface and into the substance of the facing.

4th. Cool deliberately.

5th. Grind the surface of the low-fusing addition, thoroughly removing as before the glaze, and add low-fusing body as in cases where that kind only is usually used. —*Office and Laboratory*.

**Supernumerary Teeth.** I was talking recently with a veterinarian, who told me of something that was a surprise, and may be of interest to some of my professional brothers, so I will relate it:

While the aforesaid "Hoss Doctor" was dissecting the head of a hog, he found at the angle of the jaw a sac, which on opening, he took out over fifty teeth of all sorts and sizes. He said the sac was not attached to the periosteum, but was wholly outside of it. He also informed me that this was not an uncommon condition of affairs to find in the hog. He called it merely a case of supernumerary teeth.

It might be well for our Cincinnati brethren, the next time they buy hogs' heads for head cheese, to investigate this matter.—E. H. Raffensperger, in Aug. *Summary*.

**Split Roots.** The treatment of split roots varies according to the amount of the fracture. Where the piece broken is small it is much better removed and restored with amalgam, later the edge of the amalgam under the gum can be smoothed and polished and a band crown attached. Where a single root is split down the middle the two loose pieces should be firmly tied together around the neck with floss silk. The canal should then be washed thoroughly with peroxide, dried, soaked in nitrate of silver to prevent the cracked lines from decaying, the two separate sides of the canal dovetailed and undercut, and the whole filled solidly with amalgam. When the amalgam sets the floss silk can be removed, and the root then be capped with a band crown in the ordinary way.—Dr. Head, in *Digest*.

**Vaseline and The Second Impression.** Some dentists make it a rule to vaseline the natural teeth before taking partial impressions in plaster, hoping thereby to facilitate the removal of the plaster and at the same time obtain a sharper impression. For the first impression this is rarely necessary, as the organic matter with which the teeth are normally coated is in itself somewhat oily; but for a second impression at the same sitting the vaselining of the teeth is almost a necessity in the attainment of a satisfactory result, and this simply because of the bringing away with the first impression the normal oily coating of the teeth. The smallest quantity of vaseline or its equivalent in the the meshes of a bit of cotton on the end of a hand-bur, pliers, or other suitable instrument realizes all reasonable expectations fully.—*Office and Laboratory.*

**Holocain** White crystaline powder, difficultly soluble in cold but readily so in boiling water up to two percent. Aqueous solutions neutral. Physiological action: Powerfully antiseptic and germicidal, one percent destroying the most resistant micro-organisms, preventing putrefaction and fermentation. Therapeutic indications: Ocular anesthetic. Advantages: Without influence upon width of pupil, mechanism of accommodation, croneal epitheelium or constriction of the vessels, solutions remaining sterile indefinitely. Can, however, be boiled without destroying anesthetic effect. Manner of using: One or two drops of a one percent solution instilled into the eye will produce complete anesthesia in from forty to fifty seconds. After forty seconds have elapsed, one or two drops more, and another interval of thirty seconds will find complete analgesia lasting for ten or more minutes.

**Anesthesin.** A fine white crystaline powder, almost insoluble in cold water, easily soluble in ether, alcohol, benzine and fatty oils, almond oil two percent, olive oil three percent. Solution in oil may be sterilized without decomposition. Physiological action: Local anesthetic. Therapeutic indications: Internally in gastric ulcer and hyperesthesia, nervous dyspepsia, nausea in pregnancy and in seasickness. Externally by insufflation, inhalation or painting as well as in tablets in laryngological practice, whooping-cough, tubercular and specific laryngitis

In rhinological practice as a local anesthetic. Externally to painful wounds, burns, tibial ulcer, intertrigo, pruritus from whatever cause and in eczema either as a powder or in lanolin ointment. Advantages: Absolutely nontoxic and non-irritating. Dose: Internally, five to eight grains several times daily. Externally, in powder pure or in ointment, five to twenty percent. Suppositories containing three grains each, etc.

**Sulphuric Acid and Ether for Canal Cleansing.** For the treatment of putrescent teeth and for enlarging of constricted canals, we are familiar with the Callahan method and the good results following it. In my practice I have obtained even better results from the preparation which Doctor Herbst recommends for obtunding sensitive dentin. This is prepared in the following way: A small portion of C. P. concentrated sulphuric acid is put into a glass vial, and to this is added sulphuric ether, stirring constantly with a glass rod until the acid is saturated. This is easily ascertained, for the excess of ether floats on the surfaec of the acid as a lighter colored liquid. This superfluous ether is then allowed to evaporate. Doctor Herbst also adds a few crystals of cocain hydrochlorid, and I find the addition very favorable, acting as it does as an obtundent. This chemical compound has great penetrating properties, and it is this, together with the fact that it can be quickly evaporated with warm air when desirable, which makes it so much more valuable than the sulphuric acid alone, such as Doctor Callahan uses. Its action, moreover, seems to be less irritating than the above.—A. Jessel, in *Dental Review.*

**Bridging of Spaces in Soldering.** Take of filings of an easy-flowing solder, and filings of the gold to be soldered, or some higher fusing metal, about equal parts, also borax and water rubbed up in mortar or otherwise to make a creamy solution. Mix the filings with sufficient solution to make a thick paste. Pack the joint to be soldered with this paste and heat till fused. Care should be taken that the entire mass is evenly heated throughout, and of course all the ordinary precautions of having the surfaces bright and clean, etc., must be observed. This method is especially adapted when large spaces are to be bridged, or where it is desirable to add to a cusp or to contour or otherwise change the shape of a piece in any way. Almost any form desired may be obtained, due allowance being made for shrinkage. The particles of high-fusing metal serve as a support to retain the shape of the mass, and the low-fusing solder acts as a cement when fused to unite these particles and bind them to the piece being soldered.—Dr. F. We Stephan, *Summary*.

In addition to these filings, use gold foil in strip, rop., or pellet form, packing to place with a thin plugger.—*Office and Laboratory*.

**Prophylaxis.** In an article by Dr. M. L. Rhein, in the August *Digest*, we clip the following timely suggestion. The general public has been educated to demand—a kind of mad retrograde education—the cutting or mutilating of two good sound teeth to supply the place of one lost by extraction. It seems time to sound the alarm long and loud, for should this mad course continue the last state of our patients will be worse than the first. The extraction of bad teeth to make a place for a plate made if possible for the patient to keep the mouth clean at least; but the insertion of badly-planned and constructed bridges produces a condition which makes it impracticable for the patient to have a clean and wholesome mouth. We must look to the health and comfort of our patients. The dentist must know that it is not undignified for him to remove foreign and deleterious substances from the teeth and mouth of his patients, and also use his art and skill to prevent their recurrence. We must train our eye to the cleanly and strive to secure these conditions in the mouth of our patients. If we do our part in rescuing a patient from the influence of a filthy mouth, we can trust them to do theirs to keep it so.

**Poor Old England.** Not all the Britons have surrendered to the sanitary and hygienic fads of our times. A noble little band in Folkestone, Eng., stands out resolutely against tooth-brushes. It refuses to yield to the subtle influences that are refining the Britishers into a race weaklings. At a meeting of a board of guardians of children's cottage homes a member of the board had the temerity to advocate the purchase of tooth-brushes for the children under their care. He even went so far as to declare that tooth-brushes were as necessary as soap and water.

The motion to supply tooth-brushes was vigorously opposed. The mayor of the town attacked the idea with great vehemence, declaring that the stamina of Englishmen was being ruined by such fads. He ventured the opinion that many of those present at the board meeting had not used tooth-brushes for the greater part of their lives.

And the board agreed with him, for it promptly voted down the tooth-brush proposition. The children in the cottage homes at Folkestone may not have any teeth at 45, but they will have preserved their stamina. "Back to nature" is the stirring slogan of the sturdy stalwarts of Folkestone.—*American.*

**Diagnosing for Plates.** The fact is that edentulous mouths which require artificial dentures are so unlike and varied in shape and character as to demand of the operator close study into the laws of physical force that he better apprehend the safe methods in constructing the work in hand. The edentulous mouth, into which teeth are to be inserted, should receive close study of its muscles, its repose, its play and its varied occlusion, either in over-bite or under-bite, or there will be more mistakes than Moses ever made. Take a case of mandibular protrusion and undertake to fix a denture and you have a worse problem to solve than the race question down South. Then those maxillæ with obliterated alveoli are equally grievous of mechanicalc onstruction. One who can take into his mental grasp all these conditions and evolve by his handicraft no errors is blessed above ordinary mortals in his vocation. A full denture, embodyine all the requirements of the mouth, with accurate adjustmeng of teeth in length, comeliness and occlusion, and satisfactory alike to the operator and patient, so that the critical eye cannot detect the artisan from the artist, and wherein taste and skill have been so united as to lay claim to a masterpiece of mechanism, is an achievement of which to be proud. ---Dr. Kettig, in *Digest.*

**Polishing Teeth.** I have referred to the engine and brush wheel and they have their place in dental service, but that place is not in the performance of such prophylaxis as we desire. When you polish the face of the tooth with brush wheel it will do its work so well on the face of the tooth above the gum that when we attempt to do the rest we find we have lost our guide and are deceived in doing that which is of the greater importance. I have never seen nor known of good results along this line except by the greatest care and all hand work. If the patient is sensitive to being hurt, sprinkle a little cocain salts over the gum and allow the saliva to dissolve the crystals, when you can do some vigorous service without serious complaint. The cocain should be used about the same as when applying it to adjust the rubber dam, but in no case allow the patient to swallow it. It can be used liberally in this way without danger if not swallowed. I have been cautioned by young men of the danger in using this drug, but many years' experience without the slightest symptoms of trouble has proven that it can be used judiciously in almost any case, and especially when treating gum irritations. When the mouth has been brought under perfect control there will be little if any need for the use of cocain.—Dr. Rhein.

**A New and Original Method of Making Large Porcelain Restorations.** Prepare cavity as usual for porcelain, and in making matrix be sure to anneal well if platinum foil is used. Paint walls of matrix with a solution of shellac, leaving floor free, if an approximal cavity. Let dry and fill with foundation body, to about the contour desired, leaving room, of course, for the ename' bodies of the desired shades. Bake as usual without any of the heretofore precautions, such as the cutting of grooves, inserting pieces of very high-fusing porcelain, etc. On re-removal from furnace you will find a crevice between matrix and porcelain. Now fill crevice with enamel bodies of the desired shades and finish as usual. The porcelain adheres only where the matrix is free from shellac. If the shellac is used correctly it makes it impossible for the porcelain to pull the matrix. It can be used with success in large restorations, in the anterior teeth by painting the labial half and cervical third. This helps to preserve the contour by the shrinking of the porcelain away from the matrix, instead of toward it. Then fill with enamel body the crevice and the labial portion, which has been cut away for shading. Finish as usual. The shellac burns out at a very low heat and has no effect on the porcelain whatever. To produce a clear solution, take a two-ounce bottle half full of powdered shellac, fill with alcohol and shake well. Let it stand until thoroughly clear on top, then pour off into small bottle for immediate use. The large bottle may be filled with alcohol and put aside for future use, in same manner.—G. S. Hershey, in *Review*.

**Dr. Corydon Palmer.** Dr. Corydon Palmer, who is believed to be the oldest practicing dentist in the world, was born in Warren, Ohio, in 1820. He belongs to an ancient English family who emigrated to this country in 1800. In the early part of the last century there were no colleges of dentistry and the practice was taken up by blacksmiths, wheelwrights and tinkers, and any knowledge gained by practitioners was guarded with the greatest secrecy. Dr. Palmer began life as a jeweler's apprentice, and when he turned his attention to the practice of dentistry, his skill enabled him to invent and manufacture dental instruments which were far superior to anything of the sort then used, and which were afterward used as models by the manufacturers. He was the inventor of the first set of instruments used for filling with cohesive gold. At the request of S. S. White he, years ago, examined in detail and perfected every instrument and pattern. This duty was faithfully performed and the dental profession owes a debt of gratitude to Dr. Palmer for this and many inventions. Though now in his 85th year, Dr. Palmer is still practicing dentistry in Warren, O. and manifested a lively interest in the newer methods and inventions shown at the Fourth International Dental Congress. He has two sons practicing dentistry in New York, who stand very high in the profession.

He was for a long time a member of the Board of Trustees in the Ohio Dental College at Cincinnati.

At the recent meeting of the Fourth International Dental Congress a rising vote of thanks was tendered Dr. Palmer for attending the meeting.—*American.*

**Vulcanite Shrinkage.** As the uncontrolled expansion of a 3-inch bar of vulcanite is nearly one-sixteenth of an inch at the first vulcanization, and increases with each additional vulcanization up to the fifth or sixth, by which time it reaches one-eighth inch, it follows that a plate of vulcanite changes shape very seriously in cooling. A very hard plaster will control successfully the contraction of the length and breadth of the plate, but that of its thickness does not come under the domain of the investment. I have found by experiment that a plate three-quarters inch thick over the ridge shrinks nearly one-fiftieth inch in thickness at first vulcanization. Now, as this contraction depresses the vulcanite that rests on the ridge, it acts on the fit of the plate as though it raised that of the palate, thus producing a rocking plate. Your essayist has found that the contraction of the thickness of a plate can be prevented by the use of the long-rooted teeth made for continuous-gum work, but as these are not always at hand, he employs in actual practice bars of serrated platinoid sunk vertically in the thick portions of the rubber. These can be easily held in place if inserted when the case is about three-fourths packed, and they are better if bent into forms resembling carpet tacks or the letter U, placed points downward. Another method is to drill three or four rows of pits in the model, one on the ridge and the others on the adjoining slopes. As these pits fill with rubber during flask-closing, they produce spurs of vulcanite which bind the model to the vulcanite during cooling. A second vulcanization, however, would work havoc with a plate made by this latter method, by permitting contraction, while one made with the internal serrated bars of platinoid would retain its form.—S. J. Spence, in *Digest.*

**Cocainizing Teeth Through The Nose**

Dr. O. A. LaCrone, a rhinologist of Kalamazoo, Michigan, is credited with the discovery that an adrenalin solution of cocain, on a roll of cotton, on the floor of the nose opposite the ends of the roots of the incisors and cuspids, makes possible the painless extraction and cavity preparation of all the anterior teeth and now and then bicuspids.

In his own work, which includes the sawing of bone, or its removal by other equally harsh means, he is obliged to effect complete anæsthesia of the parts, and to this end uses a six to ten percent solution of cocain with a one-tenth percent solution of adrenalin.

As a working formula for dental purposes, he offers the following:

| | | |
|---|---|---|
| Ac. Boracic | gr | iv |
| Cocain Mur | gr | xxiv |
| Adrenalin Chlor | gtt | xxiv |
| Aquae Dist | qs oz | j |

Dr. Frank W. Low, of Buffalo, N. Y., who has been chiefly instrumental in bringing this subject before the profession, finds Dr. LaCrone's formula somewhat wasteful, and substitutes Mulford Co's tablets, each of which contains of adrenalin 1-100 of a grain plus cocain 1-4 of a grain. One of these tablets in a quarter teaspoon of boiled water while still hot dissolves readily, and into this he dips a cotton plug the size of a lead pencil and about an inch long. This plug is then placed in the nostril over the roots of the teeth to be operated upon. As an addition to our limited list of methods of effecting local anæsthesia, it is bound to receive a welcome. —*Office and Laboratory.*

**Size of Porcelain Teeth.** For some inscrutable reason the manufacturers often make artificial sets of teeth having bicuspids with only two-thirds, and molars with only one-half, the area of grinding surface found in nature, and then they leave out one large molar entirely, thus reducing the occlusal area to less than half that of Mother Nature. Are we wiser than she, that we do this? It is argued by the advocates of the small molar that its lesser surface allows it to be more penetrating than the large one, and that it therefore better comminutes food. Now, this argument might be of force if man were an entirely carnivorous animal, but as he is largely a vegetable eater it is of litt'e weight, for the reason that vegetables are, as a rule, easily enough comminuted if only they can be once brought between the upper and nether millstones of the oral mill, but are quite apt to elude their grasp and be swallowed uncrushed, and of course the smaller the teeth the more is this liable to occur. Beans, corn, grape seeds and pieces of radish, celery, onions, etc., not infrequently run the gauntlet of the entire alimentary canal and escape unbroken, acting as irritants as they go. On the other hand, flesh is usually digested in the stomach, even when swallowed in large pieces. The carnivora chew their food but little; the herbivora, much. Another argument against the small bicuspid and molar is, that their inferior size prevents correct articulation. With only two small molars, instead of three large ones, it is impossible to give that curve to the line of occlusal surface which, when the mandible is protruded in biting, brings the upper and lower molars in contact at the same time that the incisors come in contact; I say, this is impossible without making said curve so abrupt that the upper plate is liable to be pushed forward in biting, as by a tilted third lower molar. The small bicuspid is also unfavorable to good articulation when the lateral bite is made, because its inner cusp has passed out and beyond the outer cusp of the opposing tooth when they ought still to remain in contact.

**National Diploma.** The endorsement of a comprehensive plan for granting a Dominion diploma in dentistry, whereby dentists could qualify for practice in any province of Canada, was a crowning feature of an entirely successful biennial convention of the Canadian Dental Association before final adjournment Sept. 8th.

The scheme, as proposed by the tentative Dominion Council and ratified by the association, contemplates the granting of certificates on passing an examination prescribed by the council, and which will be recognized by all the provincial incorporated dental associations which become parties to the agreement. British Columbia and Quebec are the only two provinces that have not yet consented, through their representatives, to the new diploma, which is to become operative on Jan. 1st next, provided the necessary provincial legislation is obtained.

According to the conditions submitted by the council, dentists who have been in practice for ten years prior to the date of their application, and who hold certificates of qualification from recognized dental colleges in Canada, will be granted the national certificate on payment of a fee. Dentists who have not been ten years in practice must pass the examination to be prescribed by the Dominion Council. Candidates applying for examinations must have a matriculation standing in arts, which matriculation standing shall not be lower than a three-years' course in a high school, with Latin as a compulsory subject.

All candidates for the diploma must have put in at least four years in dental study and have a recognized provincial diploma. Diplomas from colleges in other countries will not be recognized unless supplemented by diplomas secured in regular course from a recognized Canadian college.

The Dominion Council is to consist of two representatives from each agreeing province, who are to be elected by incorporated dental bodies, and whose term of office shall be four years.

Dr. S. W. McInnes, of Brandon, Man., in commenting on the new national diploma, declared that it would put the dental profession in Canada in the very first rank, as compared with all other countries in regard to uniform and high standards of qualification.—*American Journal.*

**Filling Came Out.** I know a very fine operator who had a *filling* come out under most embarrassing circumstances.

The case presented was a very badly-diseased antrum, being enlarged by neglect to about the size of a goose egg—or less.

It was a big cavern, to say the least, and the opening into the nose had become excessively enlarged.

It became necessary to pack it with antiseptic gauze, and a narrow roll was secured, and inserted in the usual way.

The cavern was so great that nearly the whole roll was consumed. It was pronounced as good a cotton filling as any man could make.

The patient, a man, upon being dismissed, took the car for home. After a time he felt something tickling in his nose.

He gave a sniff or two to dislodge it but without result. From a sniff he went to a snort that was so tremendous that every one craned their necks to see.

At this moment he felt and saw something fluttering on his moustache, and he thought he'd just quietly pick it off and throw it down.

He proceeded to carry out his thought—and his arm to full length. It was the cotton strip.

The other hand then took an inning; then the first, and so on, unreeling the strip.

The man was embarrassed, but couldn't stop. Some women got hysterical, and others signaled to stop and left the car. Some men laughed, and some marveled, but the patient kept busy, hand over hand.

By this time the conductor thought it time for him to "butt in." He was an Irishman. Stepping before the man he said:

"Come, now! Come, now! Phwat is goin' on here? Phwat kind of a bum wizzard are ye, anyway, to be doing yer tricks on a public conveyance? Where's your license? This is no theater, or vaudeville show. Phwy don't yees go and hire a hall and not be working the street caars wid your monkey doodle business?

"Where does yees want to get off? I'll let ye off here if ye want. Well, if ye stay on ye don't want to be reeling off any more ribbons out of your head. By the looks av that bunch on the floor your head must have been holly. When ye get off take your paraphernalia wid ye an' don't lave it behind to tangle the feet of the passengers. Put it back on yer reel if you like when ye get off the car but not in here. Wan woman was near throwin' a fit, seein' things like that; and that man in the corner was thinkin' he had the jamsey-jams. At first I thought mesilf I must have had a drink or two too much; but I'll swear to man I didn't have wan since last night whin I quit me last trip. You'll get run in the first thing you know wid your wizzard jiggery, and small worry for that on my part. If yees have anything more up your sleeve keep it up till ye get off or you'll be seeing things that are rale and that will bate yer wizzard game all to smithereens. Mind now I give ye fair warning.—Thirty-first street! Thirty-first! All right! Bing!—Bing—Bing! Thirty-second next!"—R. B. Tuller, in *American*.

